# Supplementary material for: Farmer perceptions and willingness to pay for novel livestock pest control technologies: A case of tsetse repellent collar in Kwale County in Kenya
Source: PLoS Negl Trop Dis. 2021 Aug 17;15(8):e0009663. doi: 10.1371/journal.pntd.0009663 (PMC8396722; doi:10.1371/journal.pntd.0009663)
Supplement: S1 Text — A narrative of the procedure followed to elicit the respondent’s willingness to pay for the novel tsetse repellent collar. (DOCX) [file pntd.0009663.s001.docx]

# S1 Text: Willingness to pay for a tsetse repellent collar for control of tsetse flies and Trypanosomosis transmission

Scientists in Kenya have developed repellent collars that are believed to be more effective in the control of tsetse flies than the use of trypanocidal drugs. Unlike synthetic chemicals, the collars are safe for humans and the environment. Furthermore, the collars are a one-time investment compared to the drugs that have to be applied frequently especially in high tsetse challenge areas. In addition, the collar has been identified to have multiple benefits associated with reduced tsetse fly infestation and prevalence of trypanosomosis, including reduced abortion rates, reduced animal mortality, shorter calving intervals, high milk production, the high value of oxen, efficient oxen power, and more grazing hours. One collar +repellent sachet would cost you Ksh.350 ($3.5), to be refilled every 6 weeks at approximately Ksh.125 ($1.25) (Annual cost approximately KES. 1,475) for one animal.

- - 1. How much do you use per animal/year to treat for tsetse/trypanosomosis [**BEFORE/WITHOUT ICIPE COLLAR**] (or as given in 4.4 OR KES 5,000) [**E31a]** Ksh____X___
    2. Would you buy **the repellent collar** if it is available in the **district** market and sold at the same price as your total annual expenditure (X) on animal treatment due to tsetse/trypanosomosis related health challenges [**E31b]** (X= *treatment cost without collars as given in 4.4*)*;* [____] 0=No; 1= Yes. if **YES**, go to Q 5.7.3, and if **NO** go to Q 5.7.4
    3. If **YES,** would you be willing to pay Ksh.____[**E32]***(Enumerator choose randomly any of the percent given below for the second bid and calculate the amount in monetary terms [e.g (X+(15%* X) [if* ***YES*** *or* ***No*** *go to 5.7.6]*

| **Change %** | 1. +15% | 1. +30% | 1. +45% | 1. +60% |
| --- | --- | --- | --- | --- |

- - 1. If **NO,** would you be willing to pay Ksh. ____________[**E33]** *(Enumerator* *choose randomly any of the percent given below for the second bid per and calculate the amount in monetary terms ) [e.g (X -(15% ( X] [if YES, go to 5.7.6; if* ***NO*** go to 5.7.5*]*

| **Change %** | 1. -15% | 1. -30% | 1. -45% | 1. -60% |
| --- | --- | --- | --- | --- |

- - 1. If **NOT willing to buy the repellent collar at a lower price (No** in Qnr 5.7.2& 5.7.4, why are you not willing to pay for the collar? (list three important reasons) [**E34]**

1._______________________ 2.____________________3.___________________ _

- - 1. If **YES to Q 5.7.2 & 5.7.3, when** would you be willing to buy the collar [**E35]** [_________] [***go to 5.7.7 after answering this question]***

| 1. Immediately | 1. After 1 year | 1. After 2 years | 1. After how many years? (specify)___ | 1. Other (specify)________ |
| --- | --- | --- | --- | --- |

- - 1. What size of your livestock herd would you be willing to put under repellent collars [**E36]** [*Assuming that each animal requires one collar*]

|  | Number of animals covered |  |  | Number of animals covered |
| --- | --- | --- | --- | --- |
| 1. Initial purchase [**E36a]** |  |  | 1. 2 years later [**E36c]** |  |
| 1. 1 year later **[E36b]** |  |  | 1. 3 years later **[E36d]** |  |
