# Supplementary figures and images for: Farmer perceptions and willingness to pay for novel livestock pest control technologies: A case of tsetse repellent collar in Kwale County in Kenya
Source: PLoS Negl Trop Dis. 2021 Aug 17;15(8):e0009663. doi: 10.1371/journal.pntd.0009663 (PMC8396722; doi:10.1371/journal.pntd.0009663)

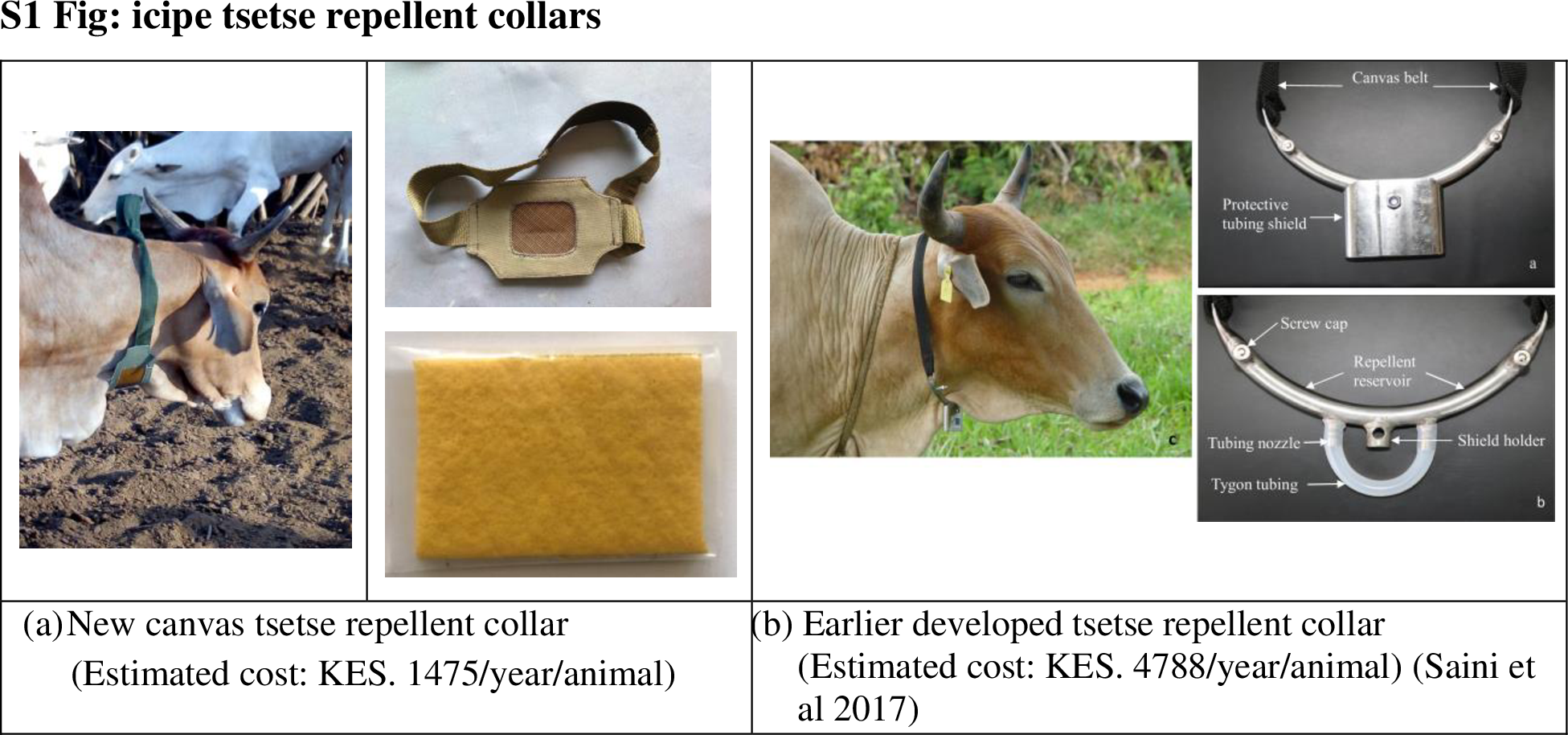

Supplement: S1 Fig — (a) New canvas tsetse repellent collar that is about to be commercialized. (b) Earlier developed tsetse repellent collar (Saini et al., 2017) [16] (TIF) [file pntd.0009663.s002.tif]
