# Supplementary material for: Farmer perceptions and willingness to pay for novel livestock pest control technologies: A case of tsetse repellent collar in Kwale County in Kenya
Source: PLoS Negl Trop Dis. 2021 Aug 17;15(8):e0009663. doi: 10.1371/journal.pntd.0009663 (PMC8396722; doi:10.1371/journal.pntd.0009663)
Supplement: S1 File — (PDF) [file pntd.0009663.s003.pdf]

```
Beatrice W. Muriithi, Gracious M. Diiro, Michael M. Kidoido, Michael O. Nyanganga, Daniel K. Masiga (2021)"Farmer Perceptions and Willingness to Pay for Novel Livestock Pest control Technologies: A case of Tsetse repellent Collar in Kwale County in Kenya"

Prepared by Dr. Beatrice Muriithii
Social Science and Impact Assessment Unit,
International Centre of Insect Physiology and Ecology
P.O. Box 30772-00100 Nairobi, Kenya
Phone: +254 (20) 8632144
Mobile: +254 (720) 663800
Email: bmuriithi@icipe.org

The aim of this file is;
1)It outlines how the variables for the above topic were constructed from the dataset obtained from farmers through a housheold level survey
2)It also shows the estimation/analysis of the willingness to pay model
3)it therefore facilitates the replication of results
4) In general, it provides a means of commnunicatoon between the author and the rest of the research community

The do file begins with construction of variables, the descriptive statistics and model estimation .



use "C:\Users\bmuriithi\Documents\Tsetse repellant literature & data\NANCY_Dr.BWM\module by module\module  2 Household composition.dta"
de,s
use "C:\Users\bmuriithi\Documents\Tsetse repellant literature & data\NANCY_Dr.BWM\module by module\module  2 Household composition.dta"
*by adult equivalent
*calculating household size in adult equivalent
*Household in Adult equivalence :OECD-modified scale" This scale, first proposed by Haagenars et al. (1994), assigns a value of 1 to the household head, of 0.5 to each additional adult member and of 0.3 to each child.
de,s
gen  adultequivalent=1 if b4_relationtohead ==1
tab adultequivalent
replace adultequivalent=0.5 if  b4_relationtohead ==2
replace adultequivalent=0.5 if b4_relationtohead ==3 & b5_age >18
*replace adultequivalent=0.5 if b4_relationtohead ==4
tab b4_relationtohead
des b4_relationtohead
label list B4
replace adultequivalent=0.5 if b4_relationtohead ==5 & b5_age >18
replace adultequivalent=0.5 if b4_relationtohead ==6 & b5_age >18
replace adultequivalent=0.5 if b4_relationtohead ==7 & b5_age >18
replace adultequivalent=0.5 if b4_relationtohead ==8 & b5_age >18
replace adultequivalent=0.5 if b4_relationtohead ==9 & b5_age >18
replace adultequivalent=0.3 if b4_relationtohead ==3 & b5_age <18
replace adultequivalent=0.3 if b4_relationtohead ==5 & b5_age <18
replace adultequivalent=0.3 if b4_relationtohead ==6 & b5_age <18
replace adultequivalent=0.3 if b4_relationtohead ==7 & b5_age <18
replace adultequivalent=0.3 if b4_relationtohead ==8 & b5_age <18
replace adultequivalent=0.3 if b4_relationtohead ==9 & b5_age <18
sum adultequivalent
des,s
edit  b4_relationtohead b5_age  adultequivalent
sort b5_age adultequivalent
replace adultequivalent=0.5 if b4_relationtohead ==4
sum adultequivalent
des,s
sort adultequivalent
edit  b4_relationtohead b5_age  adultequivalent
replace adultequivalent=0.5 if b4_relationtohead ==5 & b5_age ==18
replace adultequivalent=0.5 if b4_relationtohead ==6 & b5_age ==18
replace adultequivalent=0.5 if b4_relationtohead ==7 & b5_age ==18
replace adultequivalent=0.5 if b4_relationtohead ==8 & b5_age ==18
```

```
replace adultequivalent=0.5 if b4_relationtohead ==9 & b5_age ==18
sum adultequivalent
des,s
edit  b4_relationtohead b5_age  adultequivalent
edit  b4_relationtohead b5_age  adultequivalent
replace adultequivalent=0.5 if b4_relationtohead ==5 & b5_age==18
replace adultequivalent=0.5 if b4_relationtohead==5 & b5_age==18
edit  b4_relationtohead b5_age  adultequivalent
replace adultequivalent=0.5 if b4_relationtohead==3 & b5_age==18
des,s
sum adultequivalent
de,s
save "C:\Users\bmuriithi\Documents\Tsetse repellant literature & data\NANCY_Dr.BWM\module by module\module  2 Household composition.dta", replace
save "C:\Users\bmuriithi\Documents\Tsetse repellant literature & data\NANCY_Dr.BWM\module by module\module  2 Household composition.dta", replace
collapse (sum ) adultequivalent, by( hhid )
save "C:\Users\bmuriithi\Documents\Tsetse repellant literature & data\Data Analysis_Beatrice_from18May2020\Adult equivalent.dta"
*TLU calculations -check in the working log file- below
-------------------------------------------------------------------------------------------------------------
      name:  <unnamed>
       log:  C:\Users\bmuriithi\Documents\Tsetse repellant literature & data\Data Analysis_Beatrice_from18May2020\W
> orking log file.smcl
  log type:  smcl
 opened on:  18 May 2020, 11:54:09

. *calculating livestock TLU_owned currectly

. des c2_animatype

              storage   display    value
variable name   type    format     label      variable label
-------------------------------------------------------------------------------------------------------------
c2_animatype    long    %13.0g     animaltype
                                              Animal type

. label list   animaltype
animaltype:
           1 indegenous
           2 crossbred
           3 oxen
           4 bulls
           5 heifers
           6 calves
           7 sheep
           8 goats
           9 pig
          10 donkeys
          11 horse
          12 mule
          13 poultry
          14 rabbits
          15 other specify

. sum c4a_totalowned c4b_ownehead c4c_ownedspouse c4d_jointlyowned

    Variable |        Obs        Mean    Std. Dev.       Min        Max
-------------+---------------------------------------------------------
c4a_totalo~d |      3,028    6.720938    11.70064          1        300
c4b_ownehead |      3,028    3.941546    10.15715          0        300
c4c_owneds~e |      3,028    .3593131    2.672714          0         45
c4d_jointl~d |      3,028    2.420079    7.064563          0        200

. *calculate TLU now
```

```
122
123 . gen TLU= c4a_totalowned*0.7 if c2_animatype==1 | c2_animatype==2 | c2_animatype==3 |
    c2_animatype==4 | c2_animaty
124 > pe==5 |
125 invalid syntax
126 r(198);
127
128 . gen TLU= c4a_totalowned*0.7 if c2_animatype==1 | c2_animatype==2 | c2_animatype==3 |
    c2_animatype==4 | c2_animaty
129 > pe==5
130 (7,989 missing values generated)
131
132 . replace TLU= c4a_totalowned*0.28 if c2_animatype==6
133 (360 real changes made)
134
135 . replace TLU= c4a_totalowned*0.1 if c2_animatype==7 | c2_animatype==8
136 (571 real changes made)
137
138 . replace TLU= c4a_totalowned*0.7 if c2_animatype==10
139 (5 real changes made)
140
141 . replace TLU= c4a_totalowned*0.8 if c2_animatype==12
142 (0 real changes made)
143
144 . replace TLU= c4a_totalowned*0.2 if c2_animatype==9
145 (0 real changes made)
146
147 . replace TLU= c4a_totalowned*0.01 if c2_animatype==13
148 (588 real changes made)
149
150 . replace TLU= c4a_totalowned*0.01 if c2_animatype==14
151 (9 real changes made)
152
153 . replace TLU= c4a_totalowned*0.7 if c2_animatype==11
154 (0 real changes made)
155
156 . sum TLU
157
158     Variable |        Obs        Mean    Std. Dev.       Min        Max
159 -------------+---------------------------------------------------------
160          TLU |      3,024    1.193644    1.353308        .01       24.5
161
162 . de,s
163
164 Contains data from C:\Users\bmuriithi\Documents\Tsetse repellant literature & data\NANCY_Dr.BWM\
    module by module\mo
165 > dule 3 Livestock production.dta
166   obs:         9,480
167  vars:            15                          20 Aug 2019 12:46
168 Sorted by: c5_value
169      Note: Dataset has changed since last saved.
170
171 . edit c2_animatype c3_hhown c4a_totalowned c4b_ownehead c4c_ownedspouse c4d_jointlyowned TLU
172
173 . sort TLU
174
175 . edit hhid c2_animatype c3_hhown c4a_totalowned c4b_ownehead c4c_ownedspouse c4d_jointlyowned TLU
176
177 . edit hhid c2_animatype c3_hhown c4a_totalowned c4b_ownehead c4c_ownedspouse c4d_jointlyowned TLU
     if c4a_totalowne
178 > d>0 & TLU==.
179
180 . sort c4a_totalowned
181
182 . sort hhid
183
184 . replace c2_animatype = 1 in 511
185 (1 real change made)
```

```
186
187   . replace TLU = 7 in 511
188   (1 real change made)
189
190   . replace c2_animatype = 1 in 1664
191   (1 real change made)
192
193   . replace TLU = 4.9 in 1664
194   (1 real change made)
195
196   . replace c2_animatype = 1 in 2129
197   (1 real change made)
198
199   . replace TLU = 14 in 2129
200   (1 real change made)
201
202   . replace c2_animatype = 1 in 6742
203   (1 real change made)
204
205   . replace TLU = 2.4 in 6742
206   (1 real change made)
207
208   . save "C:\Users\bmuriithi\Documents\Tsetse repellant literature & data\NANCY_Dr.BWM\module by
      module\module 3 Live
209   > stock production.dta", replace
210   file C:\Users\bmuriithi\Documents\Tsetse repellant literature & data\NANCY_Dr.BWM\module by module
      \module 3 Livesto
211   > ck production.dta saved
212
213   . **households 12164, 21123, 23107 & 11134- these had öther specify" animal type- these were all
      replaced with indi
214   > gienous cows since they do not have other cattle types
215
216   . sum TLU
217
218       Variable |        Obs        Mean    Std. Dev.       Min        Max
219   -------------+---------------------------------------------------------
220            TLU |      3,028    1.201413     1.37815        .01       24.5
221
222   . edit hhid c2_animatype c3_hhown c4a_totalowned c4b_ownehead c4c_ownedspouse c4d_jointlyowned TLU
       if c4a_totalowne
223   > d>0 & TLU==.
224
225   . edit hhid c2_animatype c3_hhown c4a_totalowned c4b_ownehead c4c_ownedspouse c4d_jointlyowned TLU
       if c4a_totalowne
226   > d>0 & TLU==.
227
228   . sort c3_hhown
229
230   . save "C:\Users\bmuriithi\Documents\Tsetse repellant literature & data\NANCY_Dr.BWM\module by
      module\module 3 Live
231   > stock production.dta", replace
232   file C:\Users\bmuriithi\Documents\Tsetse repellant literature & data\NANCY_Dr.BWM\module by module
      \module 3 Livesto
233   > ck production.dta saved
234
235   . collapse (sum) TLU, by (hhid)
236
237   . save "C:\Users\bmuriithi\Documents\Tsetse repellant literature & data\Data
      Analysis_Beatrice_from18May2020\TLU_cu
238   > rrently.dta"
239   file C:\Users\bmuriithi\Documents\Tsetse repellant literature & data\Data
      Analysis_Beatrice_from18May2020\TLU_curre
240   > ntly.dta saved
241
242   . sum TLU
243
244       Variable |        Obs        Mean    Std. Dev.       Min        Max
```

```
-------------+--------------------------------------------------------
         TLU |        632    5.756139    3.956319        1.4       37.1

. save "C:\Users\bmuriithi\Documents\Tsetse repellant literature & data\Data
Analysis_Beatrice_from18May2020\TLU_cu
> rrently.dta", replace
file C:\Users\bmuriithi\Documents\Tsetse repellant literature & data\Data
Analysis_Beatrice_from18May2020\TLU_curre
> ntly.dta saved

. use "C:\Users\bmuriithi\Documents\Tsetse repellant literature & data\NANCY_Dr.BWM\module by
module\module 3 Lives
> tock production.dta"

. **calculate number of animals onwed by each housheold

. *start by checking if sum of c4b_ownehead+ c4c_ownedspouse+ c4d_jointlyowned= c4a_totalowned

. *egen c4a_totalowned_test=sum( c4b_ownehead c4c_ownedspouse c4d_jointlyowned)

. egen c4a_totalowned_test=rowtotal( c4b_ownehead c4c_ownedspouse c4d_jointlyowned)

. sum c4a_totalowned_test c4a_totalowned

    Variable |        Obs        Mean    Std. Dev.       Min        Max
-------------+--------------------------------------------------------
c4a_totalo~t |      9,480     2.14673    7.317069          0        300
c4a_totalo~d |      3,028    6.720938    11.70064          1        300

. sum c4a_totalowned_test c4a_totalowned if c4a_totalowned>0

    Variable |        Obs        Mean    Std. Dev.       Min        Max
-------------+--------------------------------------------------------
c4a_totalo~t |      9,480     2.14673    7.317069          0        300
c4a_totalo~d |      3,028    6.720938    11.70064          1        300

. sum c4a_totalowned_test c4a_totalowned if c4a_totalowned!=0

    Variable |        Obs        Mean    Std. Dev.       Min        Max
-------------+--------------------------------------------------------
c4a_totalo~t |      9,480     2.14673    7.317069          0        300
c4a_totalo~d |      3,028    6.720938    11.70064          1        300

. sum c4a_totalowned_test c4a_totalowned if c4a_totalowned!=.

    Variable |        Obs        Mean    Std. Dev.       Min        Max
-------------+--------------------------------------------------------
c4a_totalo~t |      3,028    6.720938    11.70064          1        300
c4a_totalo~d |      3,028    6.720938    11.70064          1        300

. **they are the same

. drop c4a_totalowned_test

. sum TLU

    Variable |        Obs        Mean    Std. Dev.       Min        Max
-------------+--------------------------------------------------------
         TLU |      3,028    1.201413     1.37815        .01       24.5

. **to calculate the number of animals owned we

. **to calculate the number of animals owned we reshape

. search reshape wide

.  reshape wide id c3_hhown c4a_totalowned c4b_ownehead c4c_ownedspouse c4d_jointlyowned c5_value
c6_totalvalue c7_
```

```
> owned7yearsago C8_totakowned7yearsago C9_value7yearsago C10_totalvalue7yrsago TLU , i(hhid) j(c2_animatype)
(note: j = 1 2 3 4 5 6 7 8 9 10 11 12 13 14 15)
values of variable c2_animatype not unique within hhid
    Your data are currently long.  You are performing a reshape wide.  You specified i(hhid) and j(c2_animatype).
    There are observations within i(hhid) with the same value of j(c2_animatype).  In the long data, variables
    i() and j() together must uniquely identify the observations.

          long                                 wide
        +---------------+                  +------------------+
        | i   j   a   b |                  | i   a1 a2  b1 b2 |
        |---------------| <--- reshape ---> |------------------|
        | 1   1   1   2 |                  | 1   1   3   2  4 |
        | 1   2   3   4 |                  | 2   5   7   6  8 |
        | 2   1   5   6 |                  +------------------+
        | 2   2   7   8 |
        +---------------+
    Type reshape error for a list of the problem variables.
r(9);

. reshape error
(note: j = 1 2 3 4 5 6 7 8 9 10 11 12 13 14 15)

i (hhid) indicates the top-level grouping such as subject id.
j (c2_animatype) indicates the subgrouping such as time.
The data are in the long form;  j should be unique within i.

There are multiple observations on the same c2_animatype within hhid.

The following 8 of 9480 observations have repeated c2_animatype values:

      +--------------------+
      |  hhid   c2_anima~e |
      |--------------------|
 511. | 11134   indegenous |
 512. | 11134   indegenous |
1651. | 12164   indegenous |
1652. | 12164   indegenous |
2116. | 21123   indegenous |
      |--------------------|
2117. | 21123   indegenous |
6736. | 23107   indegenous |
6737. | 23107   indegenous |
      +--------------------+

(data now sorted by hhid c2_animatype)

. edit hhid c2_animatype c4a_totalowned TLU if hhid==11134 | hhid== 12164 | hhid==21123 | hhid==23107

. replace c2_animatype = 15 in 511
(1 real change made)

. replace c2_animatype = 6 in 1651
(1 real change made)

. replace c2_animatype = 15 in 1657
(1 real change made)

. replace c2_animatype = 7 in 2116
(1 real change made)

. replace c2_animatype = 15 in 2123
(1 real change made)

. replace c2_animatype = 15 in 6737
```

```
(1 real change made)

. save "C:\Users\bmuriithi\Documents\Tsetse repellant literature & data\NANCY_Dr.BWM\module by module\module 3 Live
> stock production.dta", replace
file C:\Users\bmuriithi\Documents\Tsetse repellant literature & data\NANCY_Dr.BWM\module by module\module 3 Livesto
> ck production.dta saved

. save "C:\Users\bmuriithi\Documents\Tsetse repellant literature & data\NANCY_Dr.BWM\module by module\module 3 Live
> stock production.dta", replace
file C:\Users\bmuriithi\Documents\Tsetse repellant literature & data\NANCY_Dr.BWM\module by module\module 3 Livesto
> ck production.dta saved

.  reshape wide id c3_hhown c4a_totalowned c4b_ownehead c4c_ownedspouse c4d_jointlyowned c5_value c6_totalvalue c7_
> owned7yearsago C8_totakowned7yearsago C9_value7yearsago C10_totalvalue7yrsago TLU , i(hhid) j(c2_animatype)
(note: j = 1 2 3 4 5 6 7 8 9 10 11 12 13 14 15)

Data                               long   ->   wide
-----------------------------------------------------------------------------
Number of obs.                     9480   ->     632
Number of variables                  15   ->     196
j variable (15 values)     c2_animatype   ->   (dropped)
xij variables:
                                     id   ->   id1 id2 ... id15
                                c3_hhown   ->   c3_hhown1 c3_hhown2 ... c3_hhown15
                           c4a_totalowned   ->   c4a_totalowned1 c4a_totalowned2 ... c4a_totalowned15
                             c4b_ownehead   ->   c4b_ownehead1 c4b_ownehead2 ... c4b_ownehead15
                          c4c_ownedspouse   ->   c4c_ownedspouse1 c4c_ownedspouse2 ... c4c_ownedspouse15
                         c4d_jointlyowned   ->   c4d_jointlyowned1 c4d_jointlyowned2 ... c4d_jointlyowned15
                                c5_value   ->   c5_value1 c5_value2 ... c5_value15
                           c6_totalvalue   ->   c6_totalvalue1 c6_totalvalue2 ... c6_totalvalue15
                       c7_owned7yearsago   ->   c7_owned7yearsago1 c7_owned7yearsago2 ... c7_owned7yearsago15
                    C8_totakowned7yearsago   ->   C8_totakowned7yearsago1 C8_totakowned7yearsago2 ...  C8_totakowned7ye
> arsago15
                         C9_value7yearsago   ->   C9_value7yearsago1 C9_value7yearsago2 ... C9_value7yearsago15
                     C10_totalvalue7yrsago   ->   C10_totalvalue7yrsago1 C10_totalvalue7yrsago2 ... C10_totalvalue7yrs
> ago15
                                      TLU   ->   TLU1 TLU2 ... TLU15
-----------------------------------------------------------------------------

. save "C:\Users\bmuriithi\Documents\Tsetse repellant literature & data\Data Analysis_Beatrice_from18May2020\Modeul
> e 3.1_reshaped by animal type.dta"
file C:\Users\bmuriithi\Documents\Tsetse repellant literature & data\Data Analysis_Beatrice_from18May2020\Modeule 3
> .1_reshaped by animal type.dta saved

. de,s

Contains data from C:\Users\bmuriithi\Documents\Tsetse repellant literature & data\Data Analysis_Beatrice_from18May
> 2020\Modeule 3.1_reshaped by animal type.dta
  obs:           632
 vars:           196                          18 May 2020 12:36
Sorted by: hhid

. sum each of the owned livestock
```

```
variable each not found
r(111);

. search tabstat

. tabstat c4a_totalowned1  c4a_totalowned2  c4a_totalowned3  c4a_totalowned5  c4a_totalowned6  c4a_totalowned7  c4a
> _totalowned8, statistics( count mean sd min max sum )

    stats |  c4a_t~d1  c4a_t~d2  c4a_t~d3  c4a_t~d5  c4a_to~6  c4a_to~7  c4a_to~8
---------+---------------------------------------------------------------------
        N |       535        15       498       252       361       101       471
     mean |  3.166355       1.8  2.803213  2.289683  2.229917  5.257426  7.666667
       sd |   3.19298  1.897367  1.238805  1.840073  1.610656  4.232383  6.150362
      min |         1         1         1         1         1         1         1
      max |        35         8        10        16        10        25        50
      sum |      1694        27      1396       577       805       531      3611
------------------------------------------------------------------------------

. tabstat c4a_totalowned1  c4a_totalowned2  c4a_totalowned3 c4a_totalowned4  c4a_totalowned5  c4a_totalowned6  c4a_
> totalowned7  c4a_totalowned8, statistics( count mean sd min max sum )

    stats |  c4a_t~d1  c4a_t~d2  c4a_t~d3  c4a_t~d4  c4a_t~d5  c4a_to~6  c4a_to~7  c4a_to~8
---------+---------------------------------------------------------------------------------
        N |       535        15       498       193       252       361       101       471
     mean |  3.166355       1.8  2.803213  2.150259  2.289683  2.229917  5.257426  7.666667
       sd |   3.19298  1.897367  1.238805  1.487172  1.840073  1.610656  4.232383  6.150362
      min |         1         1         1         1         1         1         1         1
      max |        35         8        10        10        16        10        25        50
      sum |      1694        27      1396       415       577       805       531      3611
-------------------------------------------------------------------------------------------

. exit
-------------------------------------------------------------------------------------------------------------------
---------------
      name:  <unnamed>
       log:  C:\Users\bmuriithi\Documents\Tsetse repellant literature & data\Data Analysis_Beatrice_from18May2020\W
> orking log file.smcl
  log type:  smcl
 opened on:  18 May 2020, 12:48:51

 *TLU profiles

gen TLUprofile=0 if TLU<=3.165
replace TLUprofile=1 if TLU>3.165 & TLU<6.97
replace TLUprofile=2 if TLU>6.97
by TLUprofile, sort: sum TLU
move TLUprofile  indegenouscow
label var TLUprofile "0=low, 1=medium 3=high"
label variable TLUprofile "0=low (<=3.16), 1=medium (3.17-7.95)3=high (>6.95)"


save "C:\Users\bmuriithi\Documents\Tsetse repellant literature & data\Data Analysis_Beatrice_from18May2020\Combined variables.dta", replace
**calcualating farm size
*farm size= cultivated onwd+cultivated gift+own fallow+own grazing+homestead land
egen Ownedfarmsize=rowtotal( e37a e37b e38 e39a e40 e41)
*farm size= cultivated onwd+cultivated gift+own fallow+own grazing+homestead land+owned rented out
sum Ownedfarmsize e37a e37b e37c e38 e39a e39b e39c e40 e41
tab total_plots
edit  e37a e37b e38 e39a e40 e41 Ownedfarmsize
label var  Ownedfarmsize "=rowtotal (e37a e37b e38 e39a e40 e41)
rename Ownedfarmsize Ownedfarmsize_acres
gen Ownedfarmsize_hectare= Ownedfarmsize_acres/2.471
label var Ownedfarmsize_hectare "= Ownedfarmsize_acres/2.471
sum Ownedfarmsize_acres Ownedfarmsize_hectare
```

```
*calculate land cultivated
egen Cultivatedland_acres=rowtotal( e37a e37b e37c )
label var  Cultivatedland_acres "=rowtotal( e37a e37b e37c )
gen Cultivatedland_hectare=Cultivatedland_acres/2.471
label var Cultivatedland_hectare "=Cultivatedland_acres/2.471
sum Cultivatedland_acres Cultivatedland_hectare
edit e37a e37b e37c Cultivatedland_acres Cultivatedland_hectare
sort Cultivatedland_hectare
edit e37a e37b e37c e38- e41 Cultivatedland_acres Cultivatedland_hectare
edit hhid e37a e37b e37c e38- e41 Cultivatedland_acres Cultivatedland_hectare
gen hh_out=.
* hh_out==. potential housheolds to drop from analysis
edit hhid e37a e37b e37c e38- e41 Cultivatedland_acres Cultivatedland_hectare Ownedfarmsize_acres
Ownedfarmsize_hectare hh_out
replace hh_out = 1 in 1
replace hh_out = 1 in 2
sum Ownedfarmsize_hectare if hh_out!=., det
sum Ownedfarmsize_hectare if
hh_out==., det
gen Onwedland_profile=0 if Ownedfarmsize_hectare<=1.618778
replace Onwedland_profile=1 if Ownedfarmsize_hectare>1.618778 & Ownedfarmsize_hectare<4.046945
replace Onwedland_profile=2 if Ownedfarmsize_hectare>=4.046945
by  Onwedland_profile, sort: sum Ownedfarmsize_hectare
by  Onwedland_profile, sort: sum Ownedfarmsize_hectare if hh_out==.
label variable Onwedland_profile "0=small(<1.6"
label variable Onwedland_profile "0=small(<1.618778) 1=medium (>1.618778, <"
label variable Onwedland_profile "0=small(<1.618778) 1=medium (>1.618778, <4.046945; 2=>"
label variable Onwedland_profile "0=small(<1.618778) 1=medium (>1.618778, <4.046945; 2=>4.046945"


*off-farm income
use "C:\Users\bmuriithi\Documents\Tsetse repellant literature & data\Tsetse_data\Cleaned July
2019_for Model analysis\module 1 Household and village identification.dta", clear
use "C:\Users\bmuriithi\Documents\Tsetse repellant literature & data\Tsetse_data\Cleaned July
2019_for Model analysis\module  2 Household composition.dta"
gen off_farmincome=1 if b8_occupation==2 | b8_occupation==3 | b8_occupation==5
label var off_farmincome "=1 if b8_occupation==2 | b8_occupation==3 | b8_occupation==5
collapse (max) off_farmincome , by( hhid )
save "C:\Users\bmuriithi\Documents\Tsetse repellant literature & data\Data
Analysis_Beatrice_from18May2020\Off-farm income.dta"
sum
replace off_farmincome=0 if off_farmincome==.
sum
save "C:\Users\bmuriithi\Documents\Tsetse repellant literature & data\Data
Analysis_Beatrice_from18May2020\Off-farm income.dta", replace
**off-farm income- add income from rentals, pensions, remittances etc added to the above
edit off_farmincome m2_08 m2_09 m2_11 m2_12 m2_13
replace off_farmincome = 1 in 4
replace off_farmincome = 1 in 10
replace off_farmincome=1 if off_farmincome==0 & m2_11==1
replace off_farmincome=1 if off_farmincome==0 & m2_13==1
replace off_farmincome=1 if off_farmincome==0 & m2_08==1
replace off_farmincome=1 if off_farmincome==0 & m2_09==1
replace off_farmincome=1 if off_farmincome==0 & m2_12==1
tab off_farmincome

*************
*main occupation
des b8_occupation
label list B8
gen Mainoccupation=1 if b8_occupation==1
replace Mainoccupation=2 if b8_occupation==2
replace Mainoccupation=3 if b8_occupation==3
replace Mainoccupation=4 if b8_occupation==4 |   b8_occupation==5
label var Mainoccupation "1=Farming 2=Salaried 3=Self-employed 4=Casual"
tab Mainoccupation
*edit b8_occupation Mainoccupation
sort b8_occupation
```

```
552  edit b8_occupation Mainoccupation
553  edit b8_occupation Mainoccupation hh_out
554  gen Farmingoccupation=1 if Mainoccupation==1
555  replace Farmingoccupation=0 if Farmingoccupation==.
556  label var Farmingoccupation "1=Farming as main occupation
557  ***
558  merge 1:1 hhid using "C:\Users\bmuriithi\Documents\Tsetse repellant literature & data\Data
     Analysis_Beatrice_from18May2020\Off-farm income.dta"
559  label variable off_farmincome "(max) off_farmincome (1=yes; 0=otherwise)"
560
561  *adding distance to extension office
562  merge 1:1 hhid using "C:\Users\bmuriithi\Documents\Tsetse repellant literature &
     data\Tsetse_data\Cleaned July 2019_for Model analysis\module  2.2 infrastructure.dta"
563  order hhid - Calves b9- b19
564  sum  b14
565  save "C:\Users\bmuriithi\Documents\Tsetse repellant literature & data\Data
     Analysis_Beatrice_from18May2020\Combined variables.dta", replace
566
567
568  ******credit constrained
569  tab k2a_1
570  gen HH_neededcredit=1 if k2a_1==1 | k2a_2==1 | k2a_3==1 | k2a_4==1 | k2a_5==1 | k2a_6==1 | k2a_7==1
571  tab HH_neededcredit
572  gen Receive_credit=1 if HH_neededcredit==1 & k4_1==1
573  replace Receive_credit=1 if Receive_credit==. & HH_neededcredit==1 & k4_2==1
574  replace Receive_credit=1 if Receive_credit==. & HH_neededcredit==1 & k4_3==1
575  replace Receive_credit=1 if Receive_credit==. & HH_neededcredit==1 & k4_4==1
576  replace Receive_credit=1 if Receive_credit==. & HH_neededcredit==1 & k4_5==1
577  replace Receive_credit=1 if Receive_credit==. & HH_neededcredit==1 & k4_6==1
578  replace Receive_credit=1 if Receive_credit==. & HH_neededcredit==1 & k4_7==1
579  edit  HH_neededcredit k3a_1- k3a_7  Receive_credit
580  sort HH_neededcredit k3a_1 k3a_2 k3a_3 k3a_4 k3a_5 k3a_6 k3a_7 Receive_credit
581  replace Receive_credit = 0 in 1
582  replace Receive_credit=0 if Receive_credit==. &  HH_neededcredit==1
583  tab HH_neededcredit Receive_credit
584  egen  Receive_credit_test=rowmax( k4_1- k4_7)
585  tab  Receive_credit_test
586  drop Receive_credit_test
587  gen credit_constrained=1 if HH_neededcredit==1 & Receive_credit==0
588  tab credit_constrained
589  replace credit_constrained=0  if credit_constrained==.
590  tab credit_constrained
591  label var credit_constrained "1=Credit contrained-needed credit but didnt receive
592
593  ***generate average distance to crop and livestock main market
594  egen Distance_farmmarket=rowmean( b12 b13)
595  edit b13 b14 Distance_farmmarket
596   edit b13 b14 Distance_farmmarket
597  sum  b13 b14 Distance_farmmarket
598  drop Distance_farmmarket
599  search egen
600   egen Distance_farmmarket = rowmean( b12 b13 )
601  sum  b13 b14 Distance_farmmarket
602  edit b13 b14 Distance_farmmarket
603  egen distance_sum=rowtotal( b12 b13)
604  sum b12 b13
605  sum b12 b13 Distance_farmmarket Average_distance_sum
606  drop distance_sum Average_distance_sum
607  label var  Distance_farmmarket "= rowmean( b12 b13 )
608  label variable Distance_farmmarket "= rowmean( b12 b13 ) average distance to livestock and crop
     main markets walking '"
609
610
611  ************patricipation in livestock related rural institutions
612  tab l3a_01
613  des l3a_01
614  label list L3A
615  egen Livestock_ruralinstution=rowmax( l3a_01 l3a_02 l3b_01 l3b_02)
```

```
edit Livestock_ruralinstution  l3a_01 l3a_02 l3b_01 l3b_02
replace Livestock_ruralinstution  =0 if Livestock_ruralinstution  ==2
edit Livestock_ruralinstution  l3a_01 l3a_02 l3b_01 l3b_02
sort Livestock_ruralinstution
tab Livestock_ruralinstution
label var  Livestock_ruralinstution "=1 if household is a member of a livestick group-wife or husband
move Livestock_ruralinstution m3c
rename particepe Group_particepe

*knowledge of symptoms
***********
*gen Anorexia=1 if Other_symptoms=="Anorexia "
gen Anorexia=1 if Other_symptoms=="Anorexia "
replace Anorexia=1 if Other_symptoms=="Anorexia"
gen  Bloodyskin=1 if Other_symptoms=="Bloody  spots on the skin"
gen   Constipation =1 if Other_symptoms==" Constipation"
replace  Constipation =1 if Other_symptoms=="Constipation"
replace  Constipation =1 if Other_symptoms=="Constipation \"
replace  Constipation =1 if Other_symptoms=="Constipation "
gen   Coughing=1 if Other_symptoms=="Coughing"
replace   Coughing=1 if Other_symptoms=="Coughing "
gen   Eating_soil=1 if Other_symptoms=="Eating soil"
gen   Lacrimation_eyes=1 if Other_symptoms=="Lacrimation of eyes"
gen   Physical_weakness=1 if Other_symptoms=="Physical weakness"
replace  Physical_weakness=1 if Other_symptoms=="Physical weakness "
gen   Running_nose =1 if Other_symptoms=="Running nose "
replace   Running_nose =1 if Other_symptoms=="Running nose"
gen    Salivation =1 if Other_symptoms=="Salivation"
gen    Starring_coat =1 if Other_symptoms=="Starring coat"
replace    Starring_coat =1 if Other_symptoms=="Starring coat "
gen    Swelling_lymph=1 if Other_symptoms=="Swelling of lymph node"
save "C:\Users\bmuriithi\Documents\Tsetse repellant literature & data\Tsetse_data\Cleaned July 2019_for Model analysis\Module 3.6.4 symptoms of tsetse infestation.dta", replace
drop Starring_coat
tab Anorexia
tab Other_symptoms
tab Bloodyskin
tab Constipation
tab Coughing
tab Eating_soil
tab Lacrimation_eyes
tab Physical_weakness
tab Running_nose
tab Salivation
tab Swelling_lymph
de,s
save "C:\Users\bmuriithi\Documents\Tsetse repellant literature & data\Tsetse_data\Cleaned July 2019_for Model analysis\Module 3.6.4 symptoms of tsetse infestation.dta", replace
***generate a variable of the number of clinical symptoms that the farmer can identify
search egen
egen AATclinicalsymptoms=rowtotal( c24_abortion c24_emaciation c24_fever c24_death c24_lowmilk c24_diarrhoea c24_starringcoat c24_recumbency Anorexia Bloodyskin Constipation Coughing Eating_soil Lacrimation_eyes Physical_weakness Running_nose Salivation Swelling_lymph )
sum AATclinicalsymptoms, det
sum AATclinicalsymptoms
label var  AATclinicalsymptoms "=Total number of clinical symptoms
sum AATclinicalsymptoms
**gen Know_AATclinicalsymptoms=1 if AATclinicalsymptoms>=5
gen Know_AATclinicalsymptoms=1 if AATclinicalsymptoms>=5
replace Know_AATclinicalsymptoms=0 if Know_AATclinicalsymptoms==.
tab Know_AATclinicalsymptoms
edit AATclinicalsymptoms Know_AATclinicalsymptoms
drop AATclinicalsymptoms
egen AATclinicalsymptoms=rowtotal( c24_abortion c24_emaciation c24_fever c24_death c24_lowmilk c24_diarrhoea c24_starringcoat c24_recumbency Anorexia Bloodyskin Constipation Coughing Eating_soil Lacrimation_eyes Physical_weakness Running_nose Salivation Swelling_lymph )
label var  AATclinicalsymptoms "=Total number of clinical symptoms
```

```
sort AATclinicalsymptoms
edit Know_AATclinicalsymptoms AATclinicalsymptoms
label var Know_AATclinicalsymptoms "1=at least 5 clinical signs
gen Know_4_AATclinicalsymptoms=1 if AATclinicalsymptoms>=4
label var  Know_4_AATclinicalsymptoms "=1 if AATclinicalsymptoms>=4
tab Know_4_AATclinicalsymptoms
replace Know_4_AATclinicalsymptoms=0 if Know_4_AATclinicalsymptoms==.
tab Know_4_AATclinicalsymptoms
save "C:\Users\bmuriithi\Documents\Tsetse repellant literature & data\Tsetse_data\Cleaned July 2019_for Model analysis\Module 3.6.4 symptoms of tsetse infestation.dta", replace
sort hhid
de,s

**Tsetse prevalence
use "C:\Users\bmuriithi\Documents\Tsetse repellant literature & data\Tsetse_data\Cleaned July 2019_for Model analysis\Module 3.6.1&3.6.2 knowledge of tsetsefly.dta"
de,s
tab C17A_prevalencenow
**generate AAT prevalence
gen AAT_prevalence=1 if C17A_prevalencenow==1
tab C17B_prevalence2010
replace AAT_prevalence=0 if AAT_prevalence==.
tab AAT_prevalence
label var AAT_prevalence "1=High 0=Otherwise"
tab AAT_prevalence
save "C:\Users\bmuriithi\Documents\Tsetse repellant literature & data\Tsetse_data\Cleaned July 2019_for Model analysis\Module 3.6.1&3.6.2 knowledge of tsetsefly.dta", replace
sort hhid
save "C:\Users\bmuriithi\Documents\Tsetse repellant literature & data\Tsetse_data\Cleaned July 2019_for Model analysis\Module 3.6.1&3.6.2 knowledge of tsetsefly.dta", replace


**generating the Trypanocide effectivess- proxed for as 1=those who ranked Trypanocides as the first impprtant method for control of AAT
use "C:\Users\bmuriithi\Documents\Tsetse repellant literature & data\Tsetse_data\Cleaned July 2019_for Model analysis\Module 3.6.5 managingcontrolling tsetse.dta"
edit c25a c25a_other
sort c25a
**all those who indicated that they called a vet are classified as those who used trypanocides
tab c25a
des hhid
des c25a_other
des c25a
label list  C25A
gen Trypanocide_effec=1 if c25a==6
replace Trypanocide_effec=0 if Trypanocide_effec==.
tab Trypanocide_effec
label var Trypanocide_effec "=1 if Trypanocide is effective 0=Otherwise
save "C:\Users\bmuriithi\Documents\Tsetse repellant literature & data\Tsetse_data\Cleaned July 2019_for Model analysis\Module 3.6.5 managingcontrolling tsetse.dta", replace
de,s

*generate knowlegde on negative effects of trypanocides= proxed =1 if a farmer noted use of trypanocides as the least important AAT management strategy
gen Negetive_effct_tranocides=1 if c25c==6
sort c25a c25b c25c c25c_other
sort c25c_other
edit c25c c25c_other
replace c25c = 6 in 627
edit c25c c25c_other Negetive_effct_tranocides
replace Negetive_effct_tranocides = 1 in 627
replace c25c = 6 in 628
replace Negetive_effct_tranocides = 1 in 628
replace c25c = 6 in 413
replace c25c = 6 in 422
replace c25c = 6 in 427
replace c25c = 6 in 428
replace c25c_other = "1" in 470
```

```
737  replace Negetive_effct_tranocides = 1 in 413
738  replace Negetive_effct_tranocides = 1 in 422
739  replace Negetive_effct_tranocides = 1 in 427
740  replace Negetive_effct_tranocides = 1 in 428
741  **
742  tab Negetive_effct_tranocides
743  tab c25c
744  replace Negetive_effct_tranocides = 1 if c25c==6
745  replace Negetive_effct_tranocides=1  if Negetive_effct_tranocides==. &   c25c==6
746  replace Negetive_effct_tranocides=0 if Negetive_effct_tranocides==.
747  label var Negetive_effct_tranocides "=1 if aware of negative effects of trypanocides
748  save "C:\Users\bmuriithi\Documents\Tsetse repellant literature & data\Tsetse_data\Cleaned July
     2019_for Model analysis\Module 3.6.5 managingcontrolling tsetse.dta", replace
749
750
751  **generating WTP
752  use  "C:\Users\bmuriithi\Documents\Tsetse repellant literature & data\Tsetse_data\Cleaned July
     2019_for Model analysis\Module 5.7 Willingness to pay for an icipe collar.dta", replace
753  gen answer1= e31b
754  gen bid1= e31a
755  tab answer1
756  sum bid1
757  *gen answer2=
758  tab e32c
759  tab e33c
760  gen answer2= e32c
761  replace answer2= e33c if answer2==.
762  gen bid2= e32b
763  replace bid2= e33b if bid2==.
764  label variable answer1 "Answer to first bid "
765  label variable bid1 "Amount bid 1"
766  label variable answer2 "Answer to bid 2 (1=Yes 0=Otherwise"
767  label variable bid2 "Amount bid 2 "
768  sum answer1 bid1 answer2 bid2
769  edit answer1 bid1 answer2 bid2 e33b e33c
770  replace bid2 = . in 1
771  replace e33b = . in 1
772  replace bid2 = . in 2
773  replace bid2 = . in 3
774  replace bid2 = . in 4
775  replace answer1 = . in 1
776  replace answer1 = . in 2
777  replace answer1 = . in 3
778  replace answer1 = . in 4
779  replace answer2 = . in 1
780  replace answer2 = . in 2
781  replace answer2 = . in 3
782  replace answer2 = . in 4
783
784  doubleb bid1 bid2 answer1 answer2
785
786  initial:       log likelihood =     -<inf>  (could not be evaluated)
787  feasible:      log likelihood =  -1120.118
788  rescale:       log likelihood = -1051.8838
789  rescale eq:    log likelihood = -1022.2993
790  Iteration 0:   log likelihood = -1022.2993
791  Iteration 1:   log likelihood = -1003.8778
792  Iteration 2:   log likelihood = -1003.4582
793  Iteration 3:   log likelihood =  -1003.458
794  Iteration 4:   log likelihood =  -1003.458
795
796                                                 Number of obs     =        628
797                                                 Wald chi2(0)      =          .
798  Log likelihood =  -1003.458                    Prob > chi2       =          .
799
800  ------------------------------------------------------------------------------
801               |      Coef.   Std. Err.      z    P>|z|     [95% Conf. Interval]
802  -------------+----------------------------------------------------------------
```

```
Beta          |
       _cons |   3351.659    160.563    20.87   0.000     3036.961    3666.357
-------------+------------------------------------------------------------------
Sigma         |
       _cons |    3458.55   155.4052    22.26   0.000     3153.961    3763.139
------------------------------------------------------------------------------

First-Bid Variable:             bid1
Second-Bid Variable:            bid2
First-Response Dummy Variable:  answer1
Second-Response Dummy Variable: answer2

*mean WTP without the explanatory variables
nlcom (WTP:(_b[_cons])), noheader
. nlcom (WTP:(_b[_cons])), noheader

------------------------------------------------------------------------------
             |      Coef.   Std. Err.      z    P>|z|     [95% Conf. Interval]
-------------+----------------------------------------------------------------
         WTP |   3351.659    160.563    20.87   0.000     3036.961    3666.357
------------------------------------------------------------------------------


save "C:\Users\bmuriithi\Documents\Tsetse repellant literature & data\Tsetse_data\Cleaned July 2019_for Model analysis\Module 5.7 Willingness to pay for an icipe collar.dta", replace

*calculating mean WTP and factors that affect WTP for tsetse collars
doubleb bid1 bid2 answer1 answer2  sex_hhhead b5_age b7_education adultequivalent   TLU Ownedfarmsize_hectare Farmingoccupation off_farmincome receivtraining b14  credit_constrained Distance_farmmarket Group_participe Know_4_AATclinicalsymptoms Negetive_effct_tranocides Trypanocide_effec AAT_prevalence c36
**mean WTP
summarize sex_hhhead, meanonly
scalar sex_hhhead_m = r(mean)
summarize  b5_age, meanonly
scalar  b5_age_m = r(mean)
summarize  b7_education, meanonly
scalar  b7_education_m = r(mean)
summarize adultequivalent, meanonly
scalar adultequivalent_m = r(mean)
summarize off_farmincome, meanonly
scalar off_farmincome_m = r(mean)
summarize TLU, meanonly
scalar TLU_m = r(mean)
summarize Ownedfarmsize_hectare, meanonly
scalar Ownedfarmsize_hectare_m = r(mean)
summarize  Farmingoccupation, meanonly
scalar Farmingoccupation_m = r(mean)
summarize receivtraining, meanonly
scalar receivtraining_m = r(mean)
summarize b14, meanonly
scalar b14_m = r(mean)
summarize credit_constrained, meanonly
scalar credit_constrained_m = r(mean)
summarize Distance_farmmarket, meanonly
scalar Distance_farmmarket_m = r(mean)
summarize Group_participe, meanonly
scalar Group_participe_m = r(mean)
summarize Know_4_AATclinicalsymptoms, meanonly
scalar Know_4_AATclinicalsymptoms_m = r(mean)
summarize Negetive_effct_tranocides, meanonly
scalar Negetive_effct_tranocides_m = r(mean)
summarize Trypanocide_effec, meanonly
scalar Trypanocide_effec_m = r(mean)
summarize AAT_prevalence, meanonly
scalar AAT_prevalence_m = r(mean)
summarize  c36, meanonly
scalar c36_m = r(mean)
```

```
doubleb bid1 bid2 answer1 answer2  sex_hhhead b5_age b7_education adultequivalent   TLU Ownedfarmsize_hectare Farmingoccupation off_farmincome receivtraining b14  credit_constrained Distance_farmmarket Group_participe Know_4_AATclinicalsymptoms Negetive_effct_tranocides Trypanocide_effec AAT_prevalence c36


. doubleb bid1 bid2 answer1 answer2  sex_hhhead b5_age b7_education adultequivalent   TLU Ownedfarmsize_hectare Farmingoccupation off_farmincome receivtraining b14  credit_constrained Distance_farmmarket Group_participe Know_4_AATclinicalsymptoms Negetive_effct_tranocides Trypanocide_effec AAT_prevalence c36

initial:       log likelihood =     -<inf>  (could not be evaluated)
feasible:      log likelihood = -1530.2021
rescale:       log likelihood =  -1027.849
rescale eq:    log likelihood =  -1027.849
Iteration 0:   log likelihood =  -1027.849
Iteration 1:   log likelihood = -987.47593
Iteration 2:   log likelihood = -979.63903
Iteration 3:   log likelihood = -979.58848
Iteration 4:   log likelihood = -979.58846

                                              Number of obs     =        628
                                              Wald chi2(18)     =      48.58
Log likelihood = -979.58846                   Prob > chi2       =     0.0001

------------------------------------------------------------------------------
                             |      Coef.   Std. Err.      z    P>|z|     [95% Conf. Interval]
-----------------------------+------------------------------------------------------------------
Beta                         |
                  sex_hhhead |   839.4546   436.1599     1.92   0.054    -15.40317    1694.312
                      b5_age |  -13.05375   12.39977    -1.05   0.292    -37.35685    11.24935
                 b7_education |   70.66706   43.82991     1.61   0.107    -15.23798    156.5721
              adultequivalent |  -168.9073   158.4386    -1.07   0.286    -479.4414    141.6267
                          TLU |   11.61538    37.9844     0.31   0.760    -62.83268    86.06343
       Ownedfarmsize_hectare |   62.45497   51.97646     1.20   0.230    -39.41702     164.327
           Farmingoccupation |   48.14849   417.5981     0.12   0.908    -770.3288    866.6258
              off_farmincome |   250.2713   357.4949     0.70   0.484    -450.4057    950.9484
              receivtraining |   186.6716   398.6893     0.47   0.640    -594.7451    968.0883
                         b14 |   .7766146   1.825182     0.43   0.670    -2.800677    4.353906
          credit_constrained |   499.7167   312.4428     1.60   0.110      -112.66    1112.093
         Distance_farmmarket |   1.052686    1.29043     0.82   0.415    -1.476511    3.581882
             Group_participe |  -503.8537   451.8616    -1.12   0.265    -1389.486    381.7788
Know_4_AATclinicalsymptoms |   485.7447   387.4889     1.25   0.210    -273.7196    1245.209
 Negetive_effct_tranocides |  -55.64146   578.6202    -0.10   0.923    -1189.716    1078.433
          Trypanocide_effec |    26.5823   318.2131     0.08   0.933     -597.104    650.2686
             AAT_prevalence |   2322.183   424.4198     5.47   0.000     1490.335     3154.03
                        c36 |    385.603    558.644     0.69   0.490    -709.3192    1480.525
                      _cons |   1501.899   1417.477     1.06   0.289    -1276.305    4280.103
-----------------------------+------------------------------------------------------------------
Sigma                        |
                      _cons |   3237.411   145.6289    22.23   0.000     2951.983    3522.838
------------------------------------------------------------------------------

First-Bid Variable:             bid1
Second-Bid Variable:            bid2
First-Response Dummy Variable:  answer1
Second-Response Dummy Variable: answer2

do "C:\Users\BMURII~1\AppData\Local\Temp\STD2754_000000.tmp"
nlcom (WTP:(_b[_cons]+sex_hhhead_m*_b[sex_hhhead]+ b5_age_m*_b[ b5_age]+ b7_education_m*_b[b7_education]+adultequivalent_m*_b[adultequivalent]+off_farmincome_m*_b[off_farmincome] +TLU_m *_b[TLU]+Ownedfarmsize_hectare_m*_b[Ownedfarmsize_hectare] +Farmingoccupation_m*_b[Farmingoccupation] +receivtraining_m*_b[receivtraining] +b14_m *_b[b14] +credit_constrained_m*_b[credit_constrained] +Distance_farmmarket_m*_b[Distance_farmmarket] +Group_participe_m*_b[Group_participe] +Know_4_AATclinicalsymptoms_m*_b[Know_4_AATclinicalsymptoms] +Negetive_effct_tranocides_m*_b[Negetive_effct_tranocides] +Trypanocide_effec_m*_b[Trypanocide_effec] +AAT_prevalence_m*_b[AAT_prevalence] +c36_m*_b[c36])),  noheader
. do "C:\Users\BMURII~1\AppData\Local\Temp\STD2754_000000.tmp"
```

```
922
923  . nlcom (WTP:(_b[_cons]+sex_hhhead_m*_b[sex_hhhead]+ b5_age_m*_b[ b5_age]+ b7_education_m*_b[
     b7_education]+adultequivalent_m*_b[adultequivalent]+
924  > off_farmincome_m*_b[off_farmincome] +TLU_m *_b[TLU]+Ownedfarmsize_hectare_m*_b[
     Ownedfarmsize_hectare] +Farmingoccupation_m*_b[Farmingoccupation]
925  >  +receivtraining_m*_b[receivtraining] +b14_m *_b[b14] +credit_constrained_m*_b[
     credit_constrained] +Distance_farmmarket_m*_b[Distance_farmmarket
926  > ] +Group_participe_m*_b[Group_participe] +Know_4_AATclinicalsymptoms_m*_b[
     Know_4_AATclinicalsymptoms] +Negetive_effct_tranocides_m*_b[Negetive_e
927  > ffct_tranocides] +Trypanocide_effec_m*_b[Trypanocide_effec] +AAT_prevalence_m*_b[AAT_prevalence]
      +c36_m*_b[c36])),  noheader
928
929  ------------------------------------------------------------------------------
930               |      Coef.   Std. Err.      z    P>|z|     [95% Conf. Interval]
931  -------------+----------------------------------------------------------------
932           WTP |   3313.359   151.6254    21.85   0.000     3016.178    3610.539
933  ------------------------------------------------------------------------------
934
935  *reshaping the pests and disease section
936  **reshape wide stub, i( hhid ) j( c28 )
937  tab c28
938  tab id
939  edit
940  gen pest=1 if id=="_1"
941  replace pest=2 if id=="_2"
942  replace pest=3 if id=="_3"
943  replace pest=4 if id=="_4"
944  replace pest=5 if id=="_5"
945  tab pest
946  *reshape wide id- c30d_other , i( hhid ) j( pest )
947  reshape wide id- c30d_other , i( hhid ) j( pest )
948  tab c281
949  rename id1 id1_worms
950  rename id2 id2_Tsteste
951  rename id3 id3_Tiecks
952  rename id3_Tiecks id3_Ticks
953  rename id4 id4_smallflies
954  rename id5 id5_others
955  tab c30a_other5
956  edit id5_others- c30d_other5
957  sort c285
958  tab id1_worms
959  tab c281
960  save "C:\Users\bmuriithi\Documents\Tsetse repellant literature & data\Data
     Analysis_Beatrice_from18May2020\Module 3.6.6_reshaped.dta"
961  edit id1_worms- c30d_other1
962  tab c282
963  tab c30a2
964  tab c30a_other2
965  tab c30b2
966  tab c30b_other2
967  edit id2_Tsteste- c30d_other2
968  sum  id2_Tsteste- c30d_other2
969  label variable c282 "2 c28_does Tstetse affect your livestock "
970  save "C:\Users\bmuriithi\Documents\Tsetse repellant literature & data\Data
     Analysis_Beatrice_from18May2020\Module 3.6.6_reshaped.dta", replace
971
972  *incase the gross margins needsto be calculated; this can be done deom Module 5.1
973  . tabstat e4 e5 if e3==1, statistics( count mean sd min max ) by(E1_TECHNOLOGY)
974
975  Summary statistics: N, mean, sd, min, max
976    by categories of: E1_TECHNOLOGY (Q5.1 Animal technology)
977
978     E1_TECHNOLOGY |        e4        e5
979  -----------------+--------------------
980      Vaccinations |       359         0
981                   |   419.234         .
982                   |  1527.312         .
```

```
                    |         0         .
                    |     19200         .
------------------+--------------------
Curative (treatm |       534         0
                    |  2399.779         .
                    |  3110.808         .
                    |         0         .
                    |     30000         .
------------------+--------------------
          Natural |       359         0
                    |  6.740947         .
                    |  43.96821         .
                    |         0         .
                    |       600         .
------------------+--------------------
Artificial insem |         8         0
                    |     852.5         .
                    |   805.193         .
                    |         0         .
                    |      2500         .
------------------+--------------------
Commercial feeds |        11         0
                    |  21195.45         .
                    |  40809.94         .
                    |       250         .
                    |    131000         .
------------------+--------------------
             Salt |       155         0
                    |  667.6129         .
                    |  1476.202         .
                    |         0         .
                    |     14400         .
------------------+--------------------
         Minerals |        29         0
                    |  1359.483         .
                    |  2880.402         .
                    |         0         .
                    |     14400         .
------------------+--------------------
            Total |      1455         0
                    |  1248.991         .
                    |  4478.244         .
                    |         0         .
                    |    131000         .
-------------------------------------

use "C:\Users\bmuriithi\Documents\Tsetse repellant literature & data\Tsetse_data\Cleaned July 2019_for Model analysis\Module 9.3 Food insecurity coping strategy index.dta"
tabstat i6a i6b i6c i6d i6e, statistics( count mean mean sd min min sum )
tabstat i6a i6b i6c i6d i6e, statistics( count  mean sd min min sum )
log using "C:\Users\bmuriithi\Documents\Tsetse repellant literature & data\Data Analysis_Beatrice_from18May2020\Working log file.smcl", append
*



KAP paper revision - May 2021
*calculating confidence interval for Table 1 and 2
cii means 632 0.8386076 0.3681839
cii means 632 0.8386076 0.3681839
*gender cii means 632 0.8386076 0.3681839
*age  cii means 632 52.89241 14.09807
  cii means 632 52.89241 14.09807
*education
*age  cii means 632 7.357595 4.039658
cii means 632 7.357595 4.039658
*Household size
cii means 632 2.900475 1.003583
```

```
*Livestock TLU categories
cii means 632 2.40962 0.488249
cii means 632 4.8847 1.0685
cii means 632 10.8455 4.6383
cii means 632 5.7561 3.9563

**farm size categories
cii means 632 1.1321 0.4116
cii means 632 2.7510 0.7174
cii means 632 8.1834 3.3580
cii means 632 3.4517 3.1666

*Off-farm income
cii means 632  0.6661392 0.4719642

*Livestock training
cii means 632 1.791139 0.4068167
*Extension proximity
cii means 632 111.0301 83.4626
*Credit
cii means 632  0.4589 0.4987
*Market distance
cii means 632 215.2318 127.8820

*Rural institutions
cii means 632  0.8655 0.3415
*AAT clinical symptoms
cii means 632 0.2184 0.4135
*Negative chemical effects
cii means 632  0.0839 0.2774
*Trypanocides effectiveness
cii means 632  0.4968 0.5004
*AAT prevalence
cii means 632  0.1614 0.3682
*Aware of tsetse collar
cii means 632  0.9114 0.2844
*Main occupation
cii means 632 0.7801 0.4145

*Salaried
cii means 632 0.0997 0.2998
*Self-employed
cii means 632 0.0633 0.2437
*Casual
cii means 632 0.0443 0.2059

**Confidence interval calculations for Table 2

*Indigenous cows
cii means 535 3.17 3.19
*Exotic or cross breed
cii means 15 1.80 1.90
*Oxen
cii means 498 2.80 1.24
*Bulls
cii means 193 2.15 1.49
*Heifers
cii means 252 2.29 1.84
*Calves
cii means 361 2.23 1.61
*Sheep
cii means 101 5.26 4.23
*Goats
cii means 471 7.67 6.15
```

```
1117
1118
1119
```
