# Supplementary material for: Farmer perceptions and willingness to pay for novel livestock pest control technologies: A case of tsetse repellent collar in Kwale County in Kenya
Source: PLoS Negl Trop Dis. 2021 Aug 17;15(8):e0009663. doi: 10.1371/journal.pntd.0009663 (PMC8396722; doi:10.1371/journal.pntd.0009663)
Supplement: Attachment — Submitted filename: Review report for PLOS.docx [file pntd.0009663.s005.docx]

Review report for

Knowledge, Perceptions, and Willingness to Pay for a Tsetse Repellent Collar among Smallholder Livestock Farmers in Kwale County, Kenya

Reviewer: Dr. Daniel Kyalo Willy

April 2021

________________________________________________________________

**Abstract:**

1. The abstract needs thorough editing because several typos and sentence structure issues were noted.
2. The abstract is seemingly complete in terms of presenting elements of all the sections in the paper. However, the results on the determinants of the WTP. The authors need to provide these results as well in the abstract.

**Introduction**

- The authors mention that the “development and use of vaccines against trypanosomiasis have been futile”. This is an important statement that needs to be substantiated and/or elaborated and cited.
- The authors mention that the current study augments a study carried out 5 years prior to the current study. They mention what the other study aimed at achieving and what the current study also seeks to achieve. However, it is not clear how exactly the current study augments the previous one and therefore the gap that the current study seeks to fill is not clear. This is particularly so considering that both studies are conducted before the commercialization of the technology. The authors need to specifically explain the knowledge gap that that was missing in the previous study that the current study seeks to fill.
- **Some of the Sentences that require rephrasing include:**
- Pg 4 Line 82: “There exists evidence of drug 83 resistance as of the trypanocidal drugs……..”, The use of *as of* is improper
- Page 5, Line 108 “……. repellent to access farmers’ knowledge,attitude, and practices as regards the management of tsetse and AAT”. The aim is to **assess** not **access.**

**Materials and Methods**

- The authors start the Conceptual framework by mentioning the theoretical drivers of decision making among farmers: Utility maximization. However, this is simply given a casual mention without an elaboration on the link between the disease (as a constraint), farmers outcomes (Utility and Profits) and the choice of strategies (technology) to minimize the risk associated with ATT. What is currently presented under the Conceptual Framework section is more of Empirical Methods because the section dwells more on presenting the Contingent variation method and its application.
- Page 6 Line 136: The statement “This paper complements this previous literature in that it estimates the WTP for TRC, which has not been empirically done before.” Is misplaced and should be moved to the background section where authors present the research gap.
- On Page 10, Line 256, the Authors mention that 𝒙𝒊 is the vector of explanatory variables used to explain WTP and include proxies for human capital, labour, scale economies, access to information and services, social capital and networks, and knowledge and perceptions on tsetse and AAT management. This categorization differs from the one presented earlier on Page 9.
- There is no mention of the model that was used to estimate the determinants of WTP. As much as the authors mention that the dependent variable was linear, there is need for an explicit indication of the type of model that was used in the estimation. This issue gets confusing when going through the results presented on Table 7 and 8. It seems from these tables and the discussion on Page 17 that the same model that was used to estimate the WTP was also the same model that was used in determining factors influencing WTP.
- Once this model has been clearly indicated, which I presume is OLS, the authors also need to explain how they deal with the following econometric issues that are expected in this kind of models:
  - *Endogeinity*: Some of the explanatory variables included in this model are clearly endogenous. The farmers who have more livestock are more likely to invest in technology to protect their livestock from ATT. Therefore their WTP is obviously expected to be higher. Also, where ATT is more prevalent, livestock keepers will be more inclined to accept any technology that they feel may help them to minimize the negative effects associated with the disease.
  - *Unobserved heterogeneity*: There are several unobserved farmer attributes that are likely to be correlated with the error term.

**Results**

- The variables presented in Table 1 need to be consistent with those used in the regression model. For example, the variable: *Main occupation* is presented in the table as Categorical with 4 levels while in the model it is presented as a dummy.
- How the variable “AT clinical symptoms” was measured is not clear. Its mentioned that “Count of Four major symptoms” was used. However, it is not clear whether whatever was entered in the model was the number of clinical symptoms correctly identified (*Continuous variable*) or if a respondent identified up-to four clinical symptoms was codded 1 and the rest 0 (*Dummy variable*).
- There is need to be more insightful in the way the result are interpreted. The argument on Page 11 Line 287 that “Large household size may indicate labour availability for farm activities that may facilitate new technology” is too general. We need to see a link between these variables and the specific technology under consideration in the paper. Is the use of TRC Labour intensive? I suppose not. In that case, the consideration of the available labour at household level may not be relevant for the WTP.
- There are some statements that project the TRC technology as highly successful, which may be misleading. For instance Page 13, Line 347, the authors claim that “*On average, oxen ownership had increased by about 20% after the introduction of the icipe TRC*”. I find this kind of statement particularly misleading especially after the authors indicated in the background section that the TRC technology has not been commercialized and that it was introduced for experimental purposes. Given the lags in cattle ownership decisions, it is not convincing that a technology that is still under experimentation has causes such a substantial impact in cattle ownership (20%). Even if this happened, that authors have no way of claiming causality and attributing the increase to the introduction of the TRC technology.
- When presenting descriptive results, the authors present results that apply to general trends in Livestock production and technologies (for example see the content on sections 3.2.1 and 3.2.1). An attempt should be made to maintain focus on the objectives of the paper. Where these results have an important bearing, then this link must be established clearly.
- There is a discrepancy on the method used to estimate WTP. On Page 8, Line 181, the authors mention that this was achieved using the “Interval regression”. On Page 16, Line 235, it is mentioned that the WTP was estimated using a Logistic regression, centrally to what is presented earlier.
- Presenting the Mean WTP on Table 8 is confusing. This may appear like this was one of the explanatory variables in the model, which should not.
- The joint interpretation of the Education and Gender explanatory variables on Page 17 Line 453 in not justified. The authors should interpret each variable independently.
- The authors have avoided mentioning the magnitude of the coefficients in their interpretation of results. It is important so that the reader has an idea on the relative importance of the explanatory variables.
- Also the authors need to explain what the coefficient of the Sigma/bid variable means.

Discussion and Conclusion

- The authors have provided a summary of the results in this section. However, what we miss is a clear deduction and synthesis of the results with a clear attempt to pick out policy implications and directions on what can be done to facilitate the commercialization of the technology.
  - Given that a substantial number of the livestock keepers had low WTP for the TRC technology, how can this inform decisions on pricing of the technology?
  - What can be done to elicit sufficient interest among the commercial dealers of veterinary products?
  - Can further education and awareness creation change the WTP?

**General:**

- The paper needs further editorial work to get rid of a number of typos noted.
- The authors are inconsistent on the Citation style. For example the citation of Hanemann et al. 1991; Lusk and Hudson, 2004, on page 7 is inconsistent with the citation style used throughout the rest of paper.
