# Supplementary material for: Farmer perceptions and willingness to pay for novel livestock pest control technologies: A case of tsetse repellent collar in Kwale County in Kenya
Source: PLoS Negl Trop Dis. 2021 Aug 17;15(8):e0009663. doi: 10.1371/journal.pntd.0009663 (PMC8396722; doi:10.1371/journal.pntd.0009663)
Supplement: Attachment — Submitted filename: 21_4_26 Review CastilloCR Plos Negl.docx [file pntd.0009663.s006.docx]

REVIEW

This interesting investigation of Muriithi and Cols. is relevant for the environmental and public health, based in the previous study from icipe and by applying the contingent valuation (CV) method to elicit farmer's willingness to pay (WTP) for the novel Tsetse repellent collar (TRC) using data collected from a representative sample of cattle keepers in Kwale county, Kenya. Wide-scale commercialization and adoption of the repellent collar technology for lowing AAT transmission will depend on farmers' pre-conceived perceptions, knowledge and notably their willingness to pay, for the technology.

This technological innovation preventing disease transmission: a new paradigm for vector control. Evidence that deploying water buck repellents converts cattle into non-hosts for tsetse flies-'cows in waterbuck clothing, Saini and Cols, 2017.

Important problems:

There is not commented about limitation of study.

Refresh references. I suggest it could be important.

Observation:

1) Add the keyword: novel Tsetse repellent collar.

2)I consider important to add the effectiveness, safety

and mechanism of action of Tsetse repellent collar in the introduction.

3) It is suggested to add confidence interval in Tables 1 and 2.

4) Page 8 File 181: Which version of STATA was used?

5) I like to know about study participants: They know how to read?

If not: How answer the survey? There were excluded?

Reference:

1. Jemberu WT, Molla W, Dagnew T, Rushton J, Hogeveen H (2020) Farmers’ willingness to pay for foot and mouth disease vaccine in different cattle production systems in Amhara region of Ethiopia. PLoS ONE 15(10): e0239829. https://doi.org/10.1371/journal. pone.0239829
2. Adungo, F., Mokaya, T., Makwaga, O., & Mwau, M. (2020). Tsetse distribution, trypanosome infection rates, and small-holder livestock producers' capacity enhancement for sustainable tsetse and trypanosomiasis control in Busia, Kenya. *Tropical medicine and health*, *48*, 62. <https://doi.org/10.1186/s41182-020-00249-0>
3. [**Patterns of tsetse abundance and trypanosome infection rates among habitats of surveyed villages in Maasai steppe of northern Tanzania.**](https://pubmed.ncbi.nlm.nih.gov/28866983/) Ngonyoka A, Gwakisa PS, Estes AB, Salekwa LP, Nnko HJ, Hudson PJ, Cattadori IM.Infect Dis Poverty. 2017 Sep 4;6(1):126. doi: 10.1186/s40249-017-0340-0.
4. Olaide OY, Tchouassi DP, Yusuf AA, Pirk CWW, Masiga DK, Saini RK, Torto B. Zebra skin odor repels the savannah tsetse fly, Glossina pallidipes (Diptera: Glossinidae). PLoS Negl Trop Dis. 2019 Jun 10;13(6): e0007460. doi: 10.1371/journal.pntd.0007460. PMID: 31181060; PMCID: PMC6586361.
5. Masiga DK, Igweta L, Saini R, Ochieng'-Odero JP, Borgemeister C. Building endogenous capacity for the management of neglected tropical diseases in Africa: the pioneering role of ICIPE. PLoS Negl Trop Dis. 2014 May 15;8(5): e2687. doi: 10.1371/journal.pntd.0002687. PMID: 24830708; PMCID: PMC4022455.
