# Supplementary material for: Farmer perceptions and willingness to pay for novel livestock pest control technologies: A case of tsetse repellent collar in Kwale County in Kenya
Source: PLoS Negl Trop Dis. 2021 Aug 17;15(8):e0009663. doi: 10.1371/journal.pntd.0009663 (PMC8396722; doi:10.1371/journal.pntd.0009663)
Supplement: Attachment — Submitted filename: Responses to reveiwers comments.docx [file pntd.0009663.s007.docx]

**Responses to Reviewer’s comments**

Thank you for taking the time to read our paper and for your comments that have helped to further improve the quality of the paper. We have attempted to address your comments as much as possible. Below is a table that lists your comments and suggestions and corresponding responses.

Reviewer #1

| Serial number | Comments/inputs | Responses |
| --- | --- | --- |
| Abstract: | i)The abstract needs thorough editing because several typos and sentence structure issues were noted. | Thank you for the feedback. We have reviewed and edited the abstract for typos and grammatical errors. We hope it has improved |
|  | ii)The abstract is seemingly complete in terms of presenting elements of all the sections in the paper. However, the results on the determinants of the WTP. The authors need to provide these results as well in the abstract. | We agree with you. We missed the determinants to WTP, which we have now included. Thank you |
| Introduction | i) The authors mention that the “development and use of vaccines against trypanosomiasis have been futile”. This is an important statement that needs to be substantiated and/or elaborated and cited. | This was an oversight. Some of the previously mentioned references follow the statement [10, 14] |
|  | (ii) The authors mention that the current study augments a study carried out 5 years prior to the current study. They mention what the other study aimed at achieving and what the current study also seeks to achieve. However, it is not clear how exactly the current study augments the previous one and therefore the gap that the current study seeks to fill is not clear. This is particularly so considering that both studies are conducted before the commercialization of the technology. The authors need to specifically explain the knowledge gap that that was missing in the previous study that the current study seeks to fill. | Thank you for this observation. Conducted 5 years, the previous study was done when knowledge of AAT and Tsetse Repellant Collar (TRC) was still very limited. Besides, the type of TRC developed by then was not economically feasible for the smallholder farmers, i.e. the metallic material used to hold the repellant was too expensive for the farmers. Five (5) years later, we hypothesize that farmers' awareness (or knowledge) about AAT and TRC have significantly improved and therefore higher WTP for the technology. In addition, a new more cost-effective, and affordable material for holding and dispensing the repellent among the smallholders; subsequently this motivates the need to elicit their WTP before it is rolled out in the market. Besides, the current study utilizes contingent valuation, a utility maximization framework (Hanemann and Kanninen, 1998) that is more accurate in eliciting farmer’s WTP for the TRC compared to the conventional approach used in the previous study. We have revised the last paragraph of the introduction and hope the contribution of our study to the previous study is now clearer. |
|  | (iii) Some of the Sentences that require rephrasing include:   - Pg. 4 Line 82: “There exists evidence of drug 83 resistance as of the trypanocidal drugs…….”, The use of as of is improper - Page 5, Line 108 “……. repellent to access farmers’ knowledge, attitude, and practices as regards the management of tsetse and AAT”. The aim is to assess not access. | Thank you for the corrections; we have revised the sentences |
| Materials and Methods | (i)The authors start the Conceptual framework by mentioning the theoretical drivers of decision making among farmers: Utility maximization. However, this is simply given a casual mention without an elaboration on the link between the disease (as a constraint), farmers outcomes (Utility and Profits) and the choice of strategies (technology) to minimize the risk associated with ATT. What is currently presented under the Conceptual Framework section is more of Empirical Methods because the section dwells more on presenting the Contingent variation method and its application. | Thank you for this comment. We deliberately left out deliberate elaboration on the theory of utility maximization due to space limitations. As correctly observed, we included an introduction that justifies the use of contingent valuation. Due to space limitations, we would wish to retain this brief introduction, but we have provided a reference (Feder & Umali,1993[23]), that explains the theory of utility maximization in the context of technology adoption, such as our case. |
|  | (ii) Page 6 Line 136: The statement “This paper complements this previous literature in that it estimates the WTP for TRC, which has not been empirically done before.” Is misplaced and should be moved to the background section where authors present the research gap. | Thank you for your observation; we agree with you and have moved this statement to the introduction. |
|  | (iii) On Page 10, Line 256, the Authors mention that 𝒙𝒊 is the vector of explanatory variables used to explain WTP and include proxies for human capital, labour, scale economies, access to information and services, social capital and networks, and knowledge and perceptions on tsetse and AAT management. This categorization differs from the one presented earlier on Page 9. | Thank you for the comment. The two categories refer to the same variables, however, the categorization of information collected (Page 8-9) is broader since it captures more data than the variables mentioned on page 10. However, we have aligned them for consistency. |
|  | (iv)There is no mention of the model that was used to estimate the determinants of WTP. As much as the authors mention that the dependent variable was linear, there is need for an explicit indication of the type of model that was used in the estimation. This issue gets confusing when going through the results presented on Table 7 and 8. It seems from these tables and the discussion on Page 17 that the same model that was used to estimate the WTP was also the same model that was used in determining factors influencing WTP. | As mentioned on Page 7 (169) and Page 10 (Line 260-261), we estimated the log-likelihood (logistic) function for the double-bound WTP (Hanemann et al., 1991). This is estimated to obtain the mean WTP, first, without the explanatory variables which is the unconditional model (Results Table 7), and second, a conditional model where we include the explanatory variables (Results – Table 8). You are right, the same model estimated the results presented in the above-mentioned Tables. We followed previous studies that utilized the double-bound WTP approach such as De Groote et al, (2011) [24,25] and Kimenju et al. (2008). Also, see López-Feldman (2013) [39] on introduction to CV. |
|  | (v)Once this model has been clearly indicated, which I presume is OLS, the authors also need to explain how they deal with the following econometric issues that are expected in this kind of models:   - Endogeneity: Some of the explanatory variables included in this model are clearly endogenous. The farmers who have more livestock are more likely to invest in technology to protect their livestock from ATT. Therefore their WTP is obviously expected to be higher. Also, where ATT is more prevalent, livestock keepers will be more inclined to accept any technology that they feel may help them to minimize the negative effects associated with the disease. - Unobserved heterogeneity: There are several unobserved farmer attributes that are likely to be correlated with the error term. | Please refer to my response above; also see Page 7 (Page 169-170) for clarity of the model. Thank you  Thank you for highlighting the potential endogeneity, which we agree is a valid concern. While using primary data, we are not exclusively able to rule out endogeneity associated with certain variables such as livestock ownership. However, a test of the difference in TLU between TCR users and non-users was not significant; this also follows the regression results (Table 8) where TLU is not significant.  Similarly to our argument above, we may not be able to capture all possible unobserved farmer attributes that are likely to be correlated with the error term, however, some variables such as education capture the farmer's ability to receive and integrate new knowledge/information; indicators of social capital and knowledge and perception are also proxies for unobserved heterogeneity. This gives us confidence in our results. Moreover, the factors that are included in our model are consistent with previous studies about WTP for livestock pest and disease control products. |
| Results | (vi) The variables presented in Table 1 need to be consistent with those used in the regression model. For example, the variable: Main occupation is presented in the table as Categorical with 4 levels while in the model it is presented as a dummy. | Thank you for this observation. In Table 8, farming was compared to the other three occupations, collapsed together due to few observations as demonstrated in Table 1. We have included a note under Table 8 to explain the use of a dummy for this variable. For farm size and livestock TLU, we used quantitative measures in the model rather than categorical variables shown in Table 1. |
|  | (vii) How the variable “AT clinical symptoms” was measured is not clear. Its mentioned that “Count of Four major symptoms” was used. However, it is not clear whether whatever was entered in the model was the number of clinical symptoms correctly identified (Continuous variable) or if a respondent identified up-to four clinical symptoms was codded 1 and the rest 0 (Dummy variable). | This was as an oversight. The variable is a dummy equal to 1 if a farmer identified four clinical symptoms correctly. We have included a note under Table 8 explaining the above. Thank you |
|  | (viii) There is need to be more insightful in the way the result are interpreted. The argument on Page 11 Line 287 that “Large household size may indicate labour availability for farm activities that may facilitate new technology” is too general. We need to see a link between these variables and the specific technology under consideration in the paper. Is the use of TRC Labour intensive? I suppose not. In that case, the consideration of the available labour at household level may not be relevant for the WTP. | Thank you for the comment. Our statement is based on reviewed literature on labour availability and technology adoption. While we agree with you that TRC adoption directly is not labour intensive, indirectly the technology may increase grazing effort since the animal can now be grazed outside the homestead in tsetse infested areas such as near the forest; they can also be hired out as the technology allows more hours of land ploughing. We have added a statement on the indirect benefit of TRC (labour demand) (See Page 11 Line 291) |
|  | (ix) There are some statements that project the TRC technology as highly successful, which may be misleading. For instance Page 13, Line 347, the authors claim that “On average, oxen ownership had increased by about 20% after the introduction of the icipe TRC”. I find this kind of statement particularly misleading especially after the authors indicated in the background section that the TRC technology has not been commercialized and that it was introduced for experimental purposes. Given the lags in cattle ownership decisions, it is not convincing that a technology that is still under experimentation has causes such a substantial impact in cattle ownership (20%). Even if this happened, that authors have no way of claiming causality and attributing the increase to the introduction of the TRC technology. | This is a valid concern. However, as indicated in the introduction, although not yet commercialized, the technology has been on trial in the study area since 2011 (a decade), and over 5 years to the collection of our data. Having experienced the impact of the technology over those years, respondents had already made decisions and added their herds, while those who did not have cattle bought some. This collaborates with testimonies from farmers through qualitative information obtained from the survey area. |
|  | (x) When presenting descriptive results, the authors present results that apply to general trends in Livestock production and technologies (for example see the content on sections 3.2.1 and 3.2.1). An attempt should be made to maintain focus on the objectives of the paper. Where these results have an important bearing, then this link must be established clearly. | Thank you for this comment. Linked to your comment (ix), these trends reflect the impact of the intervention in the area over the years since the introduction of the technology in 2013. Besides, this study needs to highlight other important Livestock production constraints in the study area that may limit the uptake of TRC or whose solutions need to be integrated with those of the tsetse and AAT management. We believe these findings are important for policy, but also for other development partners that may wish to support livestock production in the area |
|  | (xi) There is a discrepancy on the method used to estimate WTP. On Page 8, Line 181, the authors mention that this was achieved using the “Interval regression”. On Page 16, Line 235, it is mentioned that the WTP was estimated using a Logistic regression, centrally to what is presented earlier. | Yes, we used interval regression in STATA to estimate the logistic (expressed) model Eq. 1 that estimates the mean WTP (López-Feldman, 2013). Please see Page 7, line 175 |
|  | (xii) Presenting the Mean WTP on Table 8 is confusing. This may appear like this was one of the explanatory variables in the model, which should not. | Thank you for the concern, however, the mean WTP is estimated using both non-conditional (Table 7) and conditional model (Table 8). The model is the same, but the conditional analysis includes socio-economic characteristics hence the mean WTP in Table 8. Also see similar results in Groote et al, (2011) and Kimenju et al. (2008). |
|  | (xiii) The joint interpretation of the Education and Gender explanatory variables on Page 17 Line 453 in not justified. The authors should interpret each variable independently. | Thank you. We have separated the explanations for the two variables. |
|  | (xiv) The authors have avoided mentioning the magnitude of the coefficients in their interpretation of results. It is important so that the reader has an idea on the relative importance of the explanatory variables. | This was deliberate since our interest was the WTP which we have explained. However, we have now included the magnitude of the coefficients following your comment. Thank you |
|  | (xv) Also the authors need to explain what the coefficient of the Sigma/bid variable means. | This is explained on page 10, the command **doubleb** uses maximum likelihood estimation to get estimates for β and Sigma (σ) that are then be used to estimate WTP (also see López-Feldman, 2013). It is for this reason that we do not explain the coefficient of the Sigma. |
| Discussion and Conclusion | (i) The authors have provided a summary of the results in this section. However, what we miss is a clear deduction and synthesis of the results with a clear attempt to pick out policy implications and directions on what can be done to facilitate the commercialization of the technology.   - Given that a substantial number of the livestock keepers had low WTP for the TRC technology, how can this inform decisions on pricing of the technology? - What can be done to elicit sufficient interest among the commercial dealers of veterinary products? - Can further education and awareness creation change the WTP? | Thank you for this observation. We have revised this section to include policy implications, this includes the need for training and awareness creation for correct diagnosis of the disease and hence correct treatment.  For the WTP, 63% of the respondents were WTP for the TRC technology at the same cost of treating an animal for AAT using trypanocidal drugs. Besides the WTP was far higher than the expected annual cost of the TRC. This result reveals a high probability of demand for the TRC.  We have added a statement on the need to sensitize commercial dealers of veterinary products, however, we are not able to provide strategies to do this since our study was limited to farmer level. This is a key area for consideration for future research.  Yes, education through training and awareness creation can enhance the WTP. We have added a sentence to reflect this in this section. |
| General | (ii) The paper needs further editorial work to get rid of a number of typos noted. | We have thoroughly edited the manuscript for typos and grammatical errors. We hope it reads better. Thank you |
|  | (iii) The authors are inconsistent on the Citation style. For example, the citation of Hanemann et al. 1991; Lusk and Hudson, 2004, on page 7 is inconsistent with the citation style used throughout the rest of paper. | This was an oversight. We have corrected the mistake and checked through the document for any other inconsistent |

**References**

1. López-Feldman (2013b). Introduction to contingent valuation using Stata. MPRA paper 41018. <https://ideas.repec.org/p/pra/mprapa/41018.html>
2. Feder, G., & Umali, D. L. (1993). The adoption of agricultural innovations: a review. Technological forecasting and social change, 43(3-4), 215-239.
3. De Groote H, Kimenju SC, Morawetz UB. Estimating consumer willingness to pay for food quality with experimental auctions: the case of yellow versus fortified maize meal in Kenya. Agric Econ. 2011;42(1):1–16.
4. Kimenju, S. C., & De Groote, H. (2008). Consumer willingness to pay for genetically modified food in Kenya. Agricultural economics, 38(1), 35-46.
5. Hanemann M, Loomis J, Kanninen B. Statistical efficiency of double-bounded dichotomous choice contingent valuation. Am J Agric Econ. 1991;73(4):1255–63.

**Reviewer #2**

| **Reviewer #2** |  |  |
| --- | --- | --- |
| General comment | This interesting investigation of Muriithi and Cols. is relevant for the environmental and public health, based in the previous study from icipe and by applying the contingent valuation (CV) method to elicit farmer's willingness to pay (WTP) for the novel Tsetse repellent collar (TRC) using data collected from a representative sample of cattle keepers in Kwale county, Kenya. Wide-scale commercialization and adoption of the repellent collar technology for lowing AAT transmission will depend on farmers' pre-conceived perceptions, knowledge and notably their willingness to pay, for the technology. This technological innovation preventing disease transmission: a new paradigm for vector control. Evidence that deploying water buck repellents converts cattle into non-hosts for tsetse flies-'cows in waterbuck clothing, Saini and Cols, 2017. | Thank you for the positive feedback on our paper. We have addressed your concerns as outlined below. |
| The objectives and hypothesis are not outlined clearly. | The study design is well explained and suitable, the selection of the respondents followed a good and robust procedure - a probability sampling was used at the last stage where the farmers were drawn from a sampling frame. The sample size is adequate, and the statistical techniques are suitable. However, there was mention of qualitative data collection and triangulation in the methods, but this was not indicated in the summary or abstract. If a mixed method was used it should appear prominently.  There are no concerns about ethical issues. | Thank you for the feedback. You are right, the study began by carrying out a qualitative survey through focus group discussions (FDGs). The objective of the FDGs was to understand the community’s knowledge and perceptions on tsetse flies, AAT, and management practices including TRC. Besides, the FDG provided information on the community profiles. This information was then utilized to design the questionnaire/tool for the household level quantitative survey that followed, as well as for validating some of the findings from the empirical analysis, hence it does not appear notably as would be if mixed methods were the study design. |
| Results | The results are well presented. However, line 491- 492 refers to 56% of respondents as majority, but this figure is about half or slightly more than half - it might be misleading therefore it should be rephrased. | Thank you for this observation; we have replaced the “majority with” a significant number” |
| Conclusions | The public health relevance should be highlighted as well as the limitations of the study. The authors have made a good case of how the study can help address the constraints of testes fly pest in livestock development. Icipe should be written in full at first time usage. | Thank you for this comment. We have highlighted the policy implications for the animal health sector (training to enhance knowledge and awareness creation and sensitizing commercial agro-dealers to stock the TRC and thus provide access to the technology to the farmers.  We have also added two limitations of our study subject for the consideration of future research |
| Editorial and Data Presentation Modifications? | I recommend the manuscript can proceed with minor revisions. A few grammatical errors appear - line 16 and 20. These should be addressed. | Thank you for the positive feedback. We have thoroughly edited the paper for grammatical and typo errors. We hope it has improved. |

**Reviewer #3**

| **Reviewer #3** |  |  |
| --- | --- | --- |
| The objectives and hypothesis are not outlined clearly. | 1.Are the objectives of the study clearly articulated with a clear testable hypothesis stated? Yes  2.-Is the study design appropriate to address the stated objectives? Yes  3.-Is the population clearly described and appropriate for the hypothesis being tested? Yes  4.-Is the sample size sufficient to ensure adequate power to address the hypothesis being tested? Yes  5.-Were correct statistical analysis used to support conclusions? Yes  6.-Are there concerns about ethical or regulatory requirements being met? No | Thank you for taking the time to read our manuscript and for the positive feedback |
| Results | Does the analysis presented match the analysis plan? Yes  -Are the results clearly and completely presented? Yes, *but it would be very convenient to add the confidence interval estimate to draw conclusions about the population characteristics from the sample results.*  -Are the figures (Tables, Images) of sufficient quality for clarity? Yes | Thank you for your observations.  We have added the confidence interval estimates (95%) in Tables 1 and 2. |
| Conclusions | -Are the conclusions supported by the data presented? Yes  -Are the limitations of analysis clearly described? No  -Do the authors discuss how these data can be helpful to advance our understanding of the topic under study? Yes  -Is public health relevance addressed? Yes | Thank you for the positive feedback |
| Editorial and Data Presentation Modifications? | No Response |  |
| Summary and General Comments | The authors have made a relevant and good case of how the study can help address the constraints of testes fly pest in livestock development. The use of a sampling fame to select respondents added rigor and strength to the study. However, the study objectives should be clearly articulated. Minor observations should be addressed. | Thank you. We have revised the last paragraph of the introduction to make the objectives of the study clearer |
| Specification | Important problems:   - There is not commented about limitation of study. - Refresh references. I suggest it could be important | Thank you for this observation. It was an oversight. We have now included the study limitation (see the last paragraph in the conclusion and recommendation sub-section).  We have refreshed the references and ensured they are consistent throughout the text |
| Observation | 1) Add the keyword: novel Tsetse repellent collar. | Thank you for the suggestion; added |
|  | 2)I consider important to add the effectiveness, safety, and mechanism of action of Tsetse repellent collar in the introduction. | This is important; we have included a statement on the same, see lines 96-98. |
|  | 3) It is suggested to add confidence interval in Tables 1 and 2. | This has been included; |
|  | 4) Page 8 File 181: Which version of STATA was used? | STATA Version 16.0 was used; we have included it in the text. |
|  | 5) I like to know about study participants: They know how to read?  If not: How answer the survey? There were excluded? | This is a valid concern. The enumerators involved in the survey spoke the same local language as the selected households to ensure none of those selected to participate in the survey was excluded and accurate data was captured. We have added a sentence in section 2.2.2 to address this concern. Thank you. |
